# T Cell Receptor Alpha Chain Genes in the Teleost Ballan Wrasse *(Labrus bergylta)* Are Subjected to Somatic Hypermutation

**DOI:** 10.3389/fimmu.2018.01101

**Published:** 2018-05-22

**Authors:** Sumaira Bilal, Kai Kristoffer Lie, Øystein Sæle, Ivar Hordvik

**Affiliations:** ^1^Department of Biological Sciences, University of Bergen, Bergen, Norway; ^2^Institute of Marine Research (IMR), Bergen, Norway

**Keywords:** T cell receptor, ballan wrasse, TCRα, polymorphism, somatic hypermutation, activation-induced cytidine deaminase motif, teleost

## Abstract

Previously, somatic hypermutation (SHM) was considered to be exclusively associated with affinity maturation of antibodies, although it also occurred in T cells under certain conditions. More recently, it has been shown that SHM generates diversity in the variable domain of T cell receptor (TCR) in camel and shark. Here, we report somatic mutations in TCR alpha chain genes of the teleost fish, Ballan wrasse (*Labrus bergylta*), and show that this mechanism adds extra diversity to the polymorphic constant (C) region as well. The organization of the TCR alpha/delta locus in Ballan wrasse was obtained from a scaffold covering a single copy C alpha gene, 65 putative J alpha segments, a single copy C delta gene, 1 J delta segment, and 2 D delta segments. Analysis of 37 fish revealed 6 allotypes of the C alpha gene, each with 1–3 replacement substitutions. Somatic mutations were analyzed by molecular cloning of TCR alpha chain cDNA. Initially, 79 unique clones comprising four families of variable (V) alpha genes were characterized. Subsequently, a more restricted PCR was performed to focus on a specific V gene. Comparison of 48 clones indicated that the frequency of somatic mutations in the VJ region was 4.5/1,000 base pairs (bps), and most prevalent in complementary determining region 2 (CDR2). In total, 45 different J segments were identified among the 127 cDNA clones, counting for most of the CDR3 diversity. The number of mutations in the C alpha chain gene was 1.76 mutations/1,000 bps and A nucleotides were most frequently targeted, in contrast to the VJ region, where G nucleotides appeared to be mutational hotspots. The replacement/synonymous ratios in the VJ and C regions were 2.5 and 1.85, respectively. Only 7% of the mutations were found to be linked to the activation-induced cytidine deaminase hotspot motif (RGYW/WRCY).

## Introduction

T cells in jawed vertebrates are generally divided into two subtypes αβ and γδ on the basis of the heterodimeric T cell receptor (TCR). The αβ T cells are most abundant in circulation and lymphoid organs, while γδ T cells are found in mucosal and epithelial tissues. In humans and mice, γδ cells represent less than 5% of the total T cell population while in birds and ruminants they constitute more than 40% of the total peripheral lymphocytes (1, 2). TCR αβ recognize peptides that are bound to major histocompatibility complex (MHC) molecules. TCR γδ recognize antigens directly, independent of MHC molecules in a manner similar to immunoglobulins (Ig). They are considered as a bridge between the innate and adaptive immune system as they use their receptor as a pattern recognition receptor (3). However, TCR γδ can also recognize phospholipids presented by CD1d molecules, suggesting that presentation by other non-classical MHC or MHC-like molecules might be possible (4). A unique TCRμ subtype first discovered in marsupials has subsequently been found in duckbill platypus (*Ornithorhynchus anatinus*), indicating that this locus was present in the last common ancestor of all extant mammals (5, 6).

T cell receptor molecules have structural and organizational resemblance to the Ig heavy and light chains. TCRα is encoded by variable (Vα) and joining (Jα) gene segments combined with the constant region (Cα) gene, like the Ig light chain. TCRβ is encoded by Vβ, diversity (Dβ), and Jβ gene segments combined with the Cβ gene, like the Ig heavy chain. Rearrangements of V, D, and J gene segments and variability generated in these junctions create an enormous repertoire of receptors with different specificities, providing versatility and diversity to the immune system (7–9). The mechanism of gene rearrangement is similar in B and T cells. V(D)J recombination is mediated by enzymes encoded by recombination activating genes 1 and 2 which recognize highly conserved recombination signal sequences (RSS). RSS are heptamer and nonamer motifs that flank the V, D, and J gene segments.

TCRα and TCRδ cDNA sequences have been reported from several teleost species, including common carp (*Cyprinus carpio*), channel catfish (*Ictalurus punctatus*), Atlantic cod (*Gadus morhua)*, rainbow trout (*Oncorhynchus mykiss*), zebrafish (*Danio rerio*), pufferfish (*Tetraodontidae rubripes, Tetraodontidae nigroviridis*, and *Sphoeroides nephelus*), Japanese flounder (*Paralichthys olivaceus*), Atlantic salmon (*Salmo salar*), and bicolor damselfish (*Stegastes partitus*) (10–20). The genomic organization of the TCRα and TCRδ genes in teleosts has been characterized in pufferfish, Atlantic salmon, and zebrafish. Like in mammals the TCRδ genes are linked to the TCRα genes, but the V gene segments are present downstream to the other elements in an inverted direction: Dδ-Jδ-Cδ-Jα-Cα-Vα/δ (15, 20–22). The Cα and Cβ sequences were considered to be relatively conserved due to interactions with other components of the TCR complex. However, allelic polymorphism of Cα and Cβ is widespread among teleost fish (17, 23).

Somatic hypermutation (SHM) is a key mechanism generating antibody diversity. In mammals, introduction of mutations in the recombined V(D)J gene in mature B cells is followed by selection of clones with higher affinities, typically in IgG-producing B-cells in germinal centers (24). Activation-induced cytidine deaminase (AID) deaminates cytosine (C) to uracil (U) in single stranded DNA creating U:G mismatch lesions, resulting in point mutations during SHM and double stranded breaks during class switch recombination. SHM occurs during transcription and primarily at RGYW/WRCY hotspot motifs (where G/C is a mutable position and R = A/G, Y = C/T, and W = A/T). SHM creates point mutations at a rate of 10^−3^ mutations/bp/generation, a million fold higher than the background genome mutation rate (25). SHM at A/T base pairs (bps) (typically WA/TW motifs) is generated by a mismatch repair mechanism employing polymerase η or other low fidelity polymerases (26). SHM has been detected in Ig genes of both cartilaginous and teleost fish (27–29). The impact of SHM on affinity maturation in fish needs further studies to be fully understood (29). Somatic mutations within Ig light chain genes of zebrafish were found to be overrepresented at AID hotspot motifs like in mammals. Mutations were most prevalent in the V region, but a significant number of substitutions were introduced in the C region; the mutation frequency decreased slightly with distance from the V region (30).

It was believed that TCR diversity was generated by V(D)J rearrangement and that SHM did not occur in the TCR genes, although SHM was detected in TCRα of mice and in TCRβ of HIV-infected patients (31, 32), and in TCR Cβ of two children after *in utero* stem cell transplantation (33). In the latter study, the frequency of Cβ mutations was significantly higher than in other groups of patients and healthy individuals (33). It was suggested that the lymphocytes of the babies presumably were under chronic activation. The SHM mechanism has also been found to target other genes, including oncogenes (34–36). A single cell PCR approach on lymph node germinal centers from a healthy person did not reveal SHM in T cells, in contrast to the situation in B cells where IgG clones were mutated (24). On the other hand, AID expression was unexpectedly detected in a subset of T cells in mice (37). More recently, studies of sandbar shark (TCRγ, TCRα) and camel (TCRγ, TCRδ) have shown that SHM occurs in TCRs of phylogenetically distant species (38–40), indicating a role in the diversification of the pre-immune repertoire. Accordingly, in mice it has been shown that early immature B cells are subjected to SHM, suggesting a role in B cell diversification as well as in the affinity maturation of antibodies (41, 42).

The aim of this study was to characterize the TCRα genes in Ballan wrasse and analyze Vα and potential Cα diversity within this species. Ballan wrasse has attracted increasing interest as a “cleaner fish” recently for the biological control of salmon lice in fish farms. Ballan wrasse belongs to *Perciformes*. Approximately 40% of all fish belong to this group (more than 10,000 species).

## Materials and Methods

### Samples

Three adult wild fish of Ballan wrasse *Labrus bergylta* (700–800 g) were caught from fjords near Bergen, Norway. Ethical approval was not required, as the study did not involve transport or experiments on live fish. Fish were killed with a sharp blow to the head immediately after they were caught and tissue samples were stored in RNA-later solution (Ambion). Previously deposited transcriptome data (intestine) from 34 juvenile fish were provided from individuals sampled in a commercial fish farm in Øygarden, Hordaland, Norway (NCBI accession numbers: PRJNA382082 and PRJNA360275).

### RNA Isolation and cDNA Synthesis

For transcriptome sequencing, intestinal tissues were homogenized using zirconium beads (4 mm) in a Precellys 24 homogenizer (Bertin Technologies, Montigny-le-Bretonneux, France) prior to RNA extraction. Total RNA was extracted using a BioRobot^®^ EZ1 and RNA Tissue Mini Kit (Qiagen, Hilden, Germany). All samples were DNase treated according to the manufacturer. RNA quality and integrity was assessed using NanoDrop ND-1000 Spectrophotometer (NanoDrop Technologies, Wilmington, DE, USA) and an Agilent 2100 Bioanalyzer with RNA 6000 Nano LabChip kit (Agilent Technologies, Palo Alto, CA, USA) respectively. The 260/280 and 260/230 nm ratios for the total RNA samples were >2.0 and the RNA integrity number >7.0 for all samples. For cDNA cloning and sequencing, total RNA was isolated from spleen and thymus using TRIzol^®^ reagent (Invitrogen). First strand cDNA was synthesized using SuperScript™ II reverse transcriptase (Invitrogen) and an oligo dT_16_ primer.

### Mapping of Intestinal Sequence Data

Raw Illumina HighSeq 2000 sequence reads deposited in the NCBI sequence read archive (SRA) database were analyzed in this study (SRA accession number: PRJNA382082). The raw FASTQ reads from individual intestinal samples originated from juvenile Ballan wrasse. Sequence adaptors were removed using Cutadapt (43, 44) with default parameters. The reads were further trimmed for low quality sequences using Sicle^1^ retaining reads with 40 bps minimum remaining sequence length and Sanger quality of 20. Prior to mapping, the quality of reads was investigated using FASTQC version 0.9.2^2^ TopHat (version 2.1.1) short read aligner and Bowtie2 (version 2.2.9) was used to individually map each sample against the *L. bergylta* genome assembly (European Nucleotide Archive accession number: PRJEB13687) (44). Subsequent BAM files were further analyzed using the IGV genome browser (version 2.3.68).

### PCR-Amplification of cDNA Fragments and DNA Sequencing

Primer construction for TCRα amplification was based on intestinal transcriptome data, genomic sequences and additional Vα sequence information obtained in the course of the present study (Table 1). Amplification using standard *Taq* polymerase (Invitrogen) was performed as follows: denaturation at 94°C for 2 min, followed by 35 cycles of denaturation at 94°C (30 s), annealing at 55°C (30 s), and extension at 72°C (1 min/1,000 bps), and final extension for 10 min. Amplification using Accuprime^TM^ *Taq* DNA polymerase and Accuprime^TM^ High Fidelity *Taq* DNA polymerase (Invitrogen) was performed as follows: denaturation at 94°C for 2 min, 30 cycles of denaturation at 94°C (30 s), annealing at 55°C (30 s), and extension at 68°C (1 min/1,000 bps). DNA fragments were excised from the gel and further amplified for 5 cycles before cloning into pCR™ 4-TOPO^®^ vector (Invitrogen). Sequencing was performed at an in-house sequencing facility using Big Dye termination chemistry (Applied Biosystems).

**Table 1:** Primers used in this study.

Primer	Primer sequence 5′→3′	Location of primers
Tcra 2F	CAGTTACAGCATCTCACCTCTACA	Leader
Tcra 2R	CCACAGTTTGAAGGTCATCAGG	TCR Cα
TCR-VF	TGGTAACACCTTGGAGGATGA	TCR Vα
TCR-JCR	CGTGCTTCTCCCTTGGTTCA	TCR Cα
TCR-CR	GCCGTCGAGTTGTTTCCCT	TCR Cα
BCD3eF1	CTAGCATCAGTGTTGGCGCT	CD3ε
BCD3eR1	CCGATGTGTGCACAGTCCTT	CD3ε

### Sequence Analysis and Phylogeny

DNA/protein sequences were compared to the GenBank/EMBL databases using BLAST.^3^ DNA was translated into amino acid sequence using the translate tool available at ExPasy.^4^ Multiple alignments were performed using ClustalW.^5^ Phylogenetic trees were constructed using MEGA6 software and neighbor joining (NJ) and maximum likelihood (ML) matrixes with 1,000 bootstrap replicates (45).

### Calculation of Mutability Index (MI) and Statistical Analysis

Mutability index is a measure of observed/expected number of mutations for a specific nucleotide without target bias. A mutability score of 1 represent unbiased mutation, while higher scores indicate that a specific nucleotide is selected for mutation. Relative frequency of each nucleotide was multiplied by total number of observed mutations within all sequenced clones to calculate the expected number of mutations. Observed numbers of mutations were divided by expected numbers for each nucleotide to calculate MI. Chi-squared analyses of MIs were carried out by comparing observed mutational frequencies to their expected (unbiased) mutational frequencies. *P* values <0.01 were considered statistically significant.

## Results

### Genomic Organization of the TCR α/δ Locus in Ballan Wrasse

**Figure 1:**
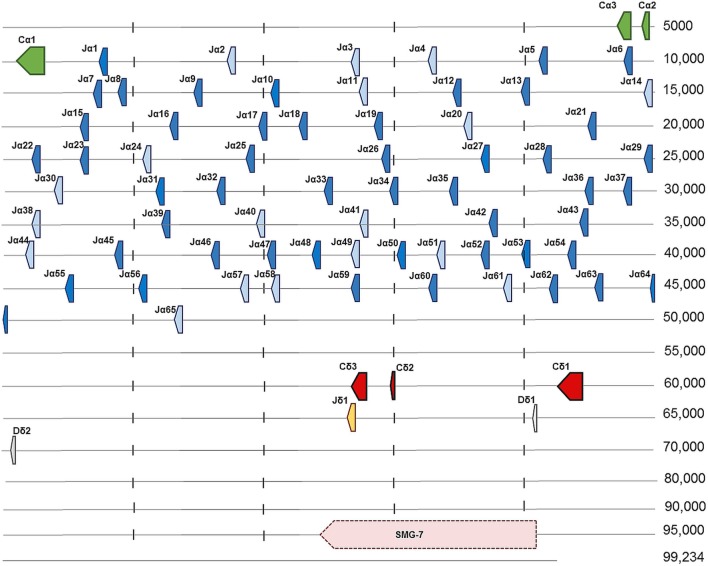
Genomic organization of the T cell receptor α/δ locus in Ballan wrasse (LaB_20160104_scaffold_928). Arrows show the direction of transcription. Cα is encoded by three exons shown in green, which are followed by 65 Jα segments. Light blue arrows present potential Jα segments, while dark blue represent the sequences found in cDNA clones. Cδ is encoded by three exons indicated here as Cδ1, Cδ2, and Cδ3. One Jδ segment is shown in yellow and two Dδ segments are represented in gray (Dδ1 and Dδ2). The SMG-7 gene was identified upstream of Cδ and its location is shown by the pink arrow.

Ballan wrasse TCRα sequences were identified by BLAST searches in an intestine transcriptome database using salmon TCRα as query (18). In the course of the present work, genomic sequence data of a heterozygous individual became available, and two scaffolds containing two allelic variants of the Cα gene in Ballan wrasse were identified by BLAST searches of whole genome shotgun data (GenBank): LaB_20160104_scaffold_928 (99,234 nt) and LaB_20160104_scaffold_4467 (16,780 nt). The Cα gene consisted of three exons corresponding to the Ig domain, the connecting peptide (CP) and the transmembrane (TM)/cytoplasmic (CYT) part. In total, 65 putative Jα segments were found by manual inspection of the region upstream of Cα in scaffold 928. All putative Jα segments contained the highly conserved core motif FGXG or slightly modified versions of this, and splice sites and RSS flanking the Jα exons. The presence of J segments was further confirmed by alignment of transcriptome data with scaffold 928, using the IGV program (46). The TCRα cDNA clones characterized in this study contained 45 of the 65 identified Jα segments. A single Jδ segment and two putative Dδ segments were identified upstream of the Cδ gene. As in other teleosts, the SMG-7 gene was identified further upstream of Cδ (Figure 1; Table S1 in Supplementary Material). Scaffold 4467 was shorter and represented the other allele of the TCRα locus, comprising the Cα gene and 13 Jα segments (Table S2 in Supplementary Material). Several scaffolds containing Vα genes were found in the genomic sequence database, but a complete assembly of all Vα genes was not possible based on the present whole genome shotgun data.

### Sequence Analysis of TCRα cDNA Clones

**Figure 2:**
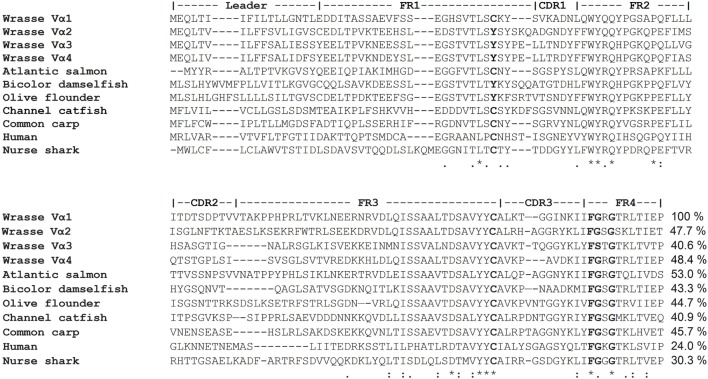
Representatives of four TCRα V gene families from Ballan wrasse aligned with corresponding sequences from other species. Amino acid percentage identities between Ballan wrasse Vα1 and the other sequences are indicated at the end. GenBank accession numbers are: Wrasse Vα1 (MG594685), Wrasse Vα2 (MG594679), Wrasse Vα3 (MG594717), Wrasse Vα4 (MG594664), Atlantic salmon (ABO72169.1), bicolor damselfish (AAO88984.1), olive flounder (BAB82535.1), common carp (BAD88980.1), channel catfish (AAD56889.1), nurse shark (ADW95871.1), and human (AAD15154.1).

The assembled Ballan wrasse TCRα sequence was used as a basis for primer construction, and cDNAs encoding part of the leader sequence, V/J, and Cα were amplified by PCR. In total, 79 distinct TCRα clones were analyzed from three individuals. The V gene regions of the cDNAs were sorted into four groups based on 75% nucleotide identity (Figure S1 in Supplementary Material). The translated sequences in group Vα1 showed all conserved characteristics of a V domain, while Vα2, Vα3, and Vα4 lacked the conserved cysteine (Cys) at position 26 (replaced with tyrosine); a pattern which was also seen in bicolor damselfish and olive flounder (16, 17). Figure 2 shows representatives of the four Vα families from Ballan wrasse aligned with TCR Vα sequences of other species. The Vα1 amino acid sequences have identity indices of 40–48% with the other characterized groups in wrasse; Vα2, Vα3, and Vα4. Among other species, Vα1 has 53% sequence identity to Atlantic salmon followed by common carp (45.7%) and olive flounder (44.7%).

**Figure 3:**
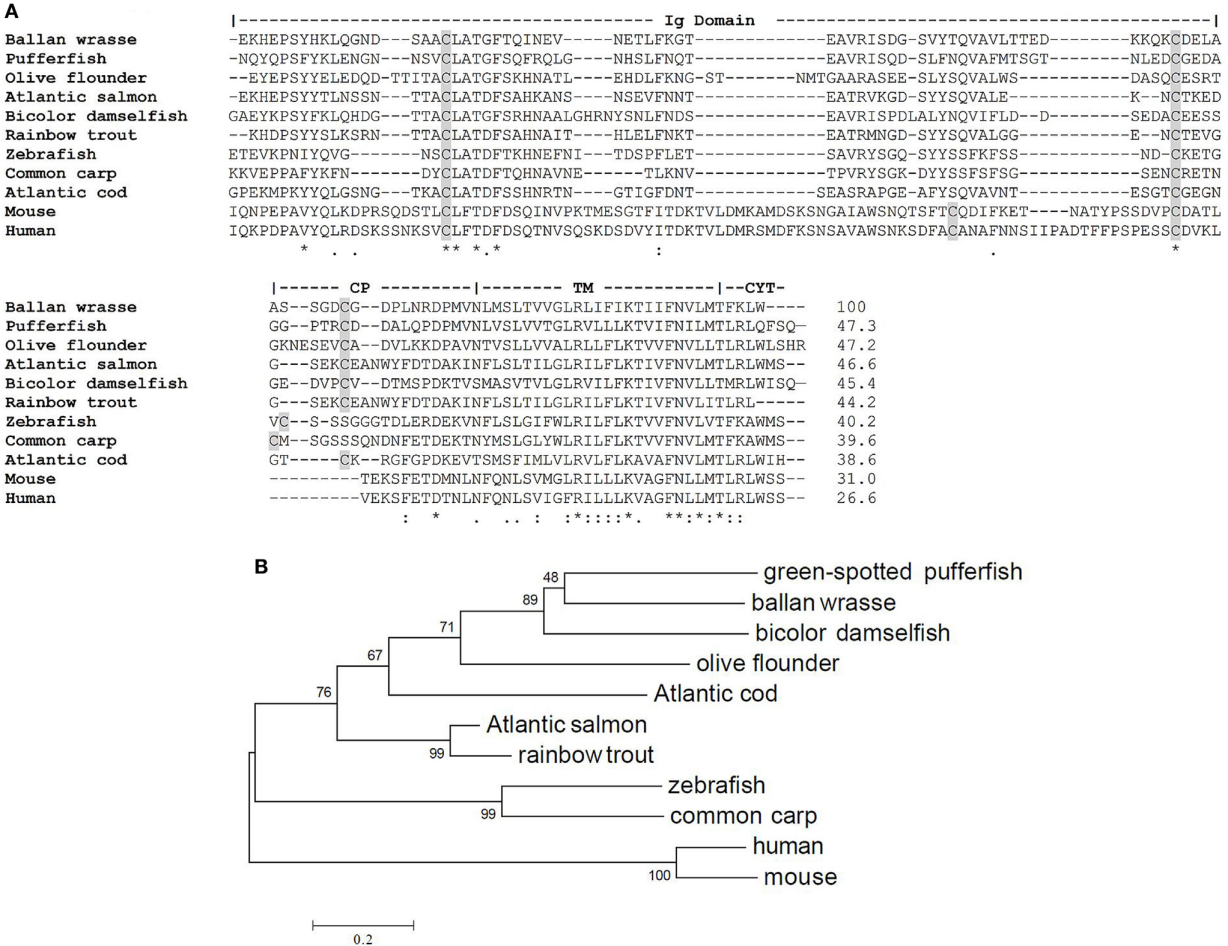
Similarity between T cell receptor (TCR) Cα of Ballan wrasse and other species. **(A)** Alignment of TCR Cα sequences from Ballan wrasse (allotype A) and other species. Cysteines are shaded in gray. **(B)** Phylogenetic tree of Ballan wrasse TCR Cα and other species. The unrooted tree was constructed by the neighbor joining method with 1,000 bootstrap replicates using MEGA6 software. The accession numbers are as follows: Green-spotted pufferfish (CAC86243.1), bicolor damselfish (AAO88997.1), olive flounder (BAC65457.1), Atlantic salmon (AAS79492.1), rainbow trout (AAR19288.1), Atlantic cod (CAD28810.1), common carp (BAD89003.1), zebrafish (AAL29402.1), mouse (X14387.1), and human (L02424.1).

Wrasse TCR Cα encodes a polypeptide of 112 amino acids. The Cα region can be divided into an Ig domain, CP, TM, and CYT part. The structurally important Cys residues in the Ig domain and CP are conserved. The TM region is the most conserved region containing the positively charged residues lysine and arginine involved in the assembly of the TCR–CD3 complex. Multiple sequence alignment demonstrated that Ballan wrasse TCR Cα has 47.3% sequence identity to pufferfish, 46.6% with salmon, 40.2% with zebrafish, and 38.6% with cod, 31% with mouse, and 26.6% with human (Figure 3A). The phylogenetic relationship between Ballan wrasse Cα and the orthologous molecules in other species is shown in Figure 3B.

### Cα Polymorphism

**Figure 4:**
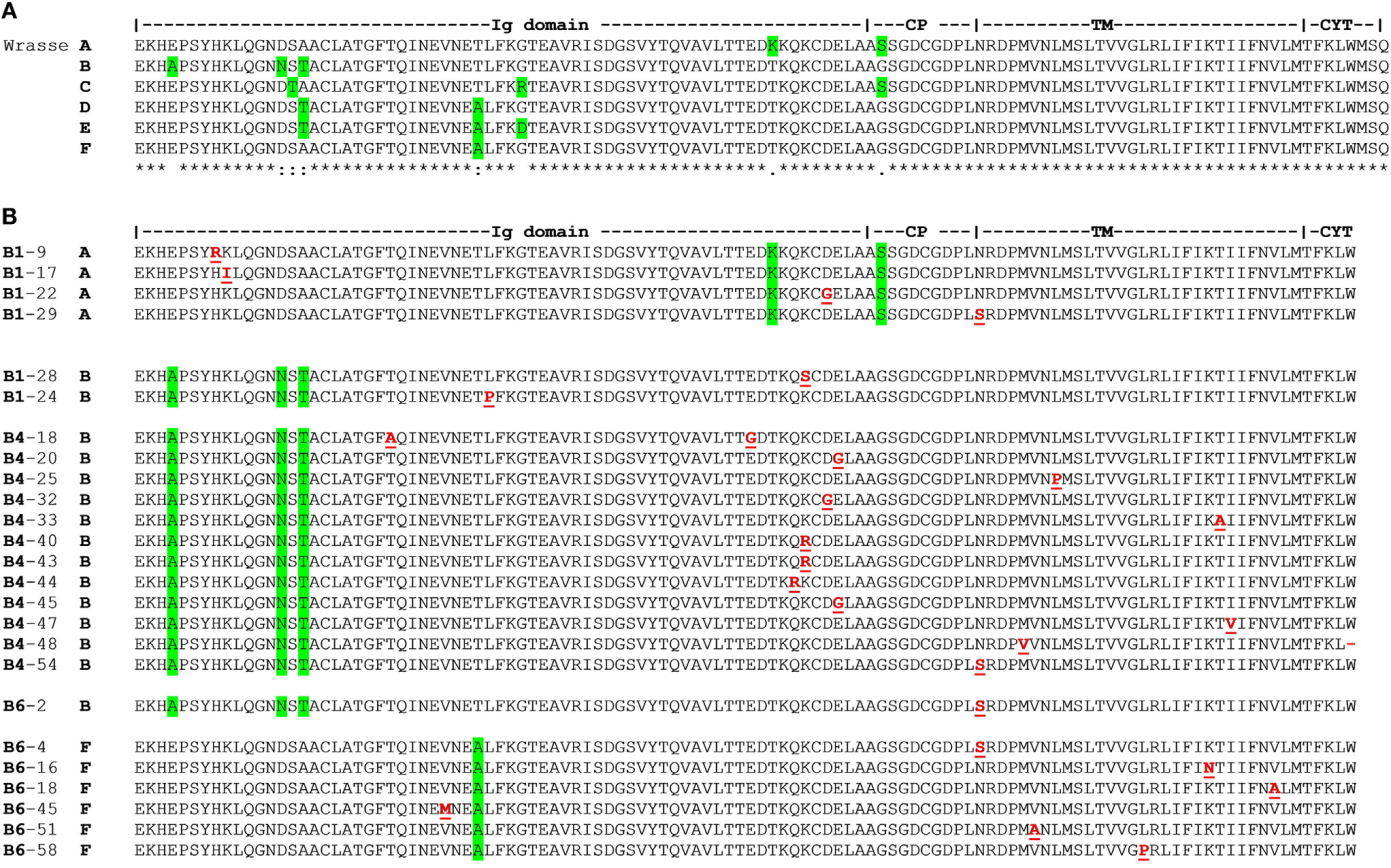
T cell receptor (TCR) Cα allotypes and somatic replacement mutations in Ballan wrasse. **(A)** TCR Cα allotypes in Ballan wrasse. Allotypes were found by analysis of scaffold 928 and 4467, intestinal transcriptome data from 34 Ballan wrasse individuals, and 79 cDNA clones from three wild fish (B1, B4, and B6). Green highlights residues which characterize each allotype. **(B)** Alignment and positioning of somatic replacement mutations found in TCR Cα of individuals B1, B4, and B6. Mutations are underlined in red. Accession numbers are given in Table S10 in Supplementary Material.

The two scaffolds 928 and 4467 represent distinct Cα alleles; here named A and B. Analysis of transcriptome data from 34 farmed individuals identified A and B, and four additional allotypes of Cα named C, D, E, and F. Molecular cloning of TCRα cDNA from three wild fish corresponded to alleles A, B, and F [B1: (A/B), B4: (B/B), and B6: (B/F); Figure 4A; Table S3 in Supplementary Material].

### Cα Somatic Mutations

Molecular cloning of TCRα from thymus cDNA of individual B1 showed that there was a significant number of point mutations in Cα (1.75/1,000 bps). Amplification of the first sample was done with standard *Taq* polymerase and 5,690 bps were sequenced (18 cDNA clones). In a second experiment, TCRα from spleen cDNA was amplified from individual B4 with high fidelity *Taq* polymerase. As a control, CD3ε cDNA was amplified under the exact same conditions. The frequency of mutations in Cα was 2.30/1,000 bps versus 0.62/1,000 bps in CD3ε. In total, 6,952 bps of Cα (22 cDNA clones) and 12,992 bps of CD3ε were analyzed. In a third experiment, TCRα was amplified from spleen cDNA with high fidelity *Taq* polymerase from individual B6, revealing 1.46 mutations/1,000 bps in a total of 12,327 bps (39 cDNA clones) (Figure 4B; Table 2). All cDNA clones examined were confirmed to be unique, possessing distinct V regions. Two clones were regarded to be artifacts of PCR jumping (i.e., partial extension on cDNA from one gene and final extension on another, resulting in hybrids of two alleles). The replacement to synonymous mutation ratio (R/S) was 1.85 and most replacements were conservative (i.e., the biochemical properties were not changed; hydrophobic, charged, and neutral, etc.). In total, 93% of the nucleotide substitutions were transitions (primarily A to G and T to C). Of all mutations, 37.5% were targeted at WA motifs, whereas 7.5% were targeted at AID motifs. Three WA motifs were present in WRCY (AID hotspot), but the targeted nucleotide was not at the C position (Table S4 in Supplementary Material).

**Table 2:** Cα-mutations in 79 cDNA clones of three Ballan wrasse individuals.

Name of individual fish	# Clones	# Base pairs (bps)	# Mut	Mut/1,000 bps	Types of mutations				
					Repl.	Silent	Transition	Transversion	Frameshift
B1	18	5,690	10	1.75	6	2	7	1	2
B4	22	6,952	16	2.30	12	4	16	0	0
B6	39	12,327	18	1.46	8	8	14	2	2
Total	79	24,969	44	1.76	26	14	37	3	4

### Mutability Indices for Mono, Di, and Trinucleotides Indicate Targets for Cα Mutation

To determine which nucleotides or combinations of adjacent nucleotides were preferentially targeted for mutation in the Cα gene, MIs for mono, di, and trinucleotides were calculated. Chi-square analysis showed that only A nucleotides were significantly targeted for mutation, 93% of which were transitions. C nucleotides were significant cold spots for mutation. MI scores showed that nucleotides were targeted in the order: A > T > G > C for somatic mutations (Table 3). The dinucleotide MIs revealed that AA, AT, GA, and AG were significant targets for mutation and when the analysis was expanded to trinucleotides, the dinucleotides were most frequently targeted in AAT, AAA, and AGA combinations (Tables S5 and S6 in Supplementary Material). This pattern of substitution indicates that mutations targeted particular nucleotides and combinations.

**Table 3:** Substitutions and mutability index (MI) of T cell receptor (TCR) Cα mononucleotides.

From	Substitutions				Observed mutations	Expected mutations	MI
	A	C	G	T			
A	–	1	25	1	27	10.6	2.5^a^
C	0	–	0	1	1	11	0.09^b^
G	5	1	–	0	6	10	0.6
T	0	8	0	–	8	8.2	0.97

*In total, 26,862 TCR Cα nucleotides were analyzed (A = 7109, T = 5528, G = 6846, C = 7379). There were 40 point mutations. MI values were calculated by dividing observed number of mutations to expected number of mutations. The observed and expected numbers of mutations were compared by χ^2^ analysis and significant differences are indicated on MI values*.

*^a^Statistically significant by χ^2^ test (*p* < 0.001)*.

*^b^Statistically significant by χ^2^ test (*p* < 0.01)*.

### Vα Somatic Mutations

To study the mutation pattern in one single Vα gene, the B4-34 sequence was selected, and primers (TCR-VF/TCR-CR) were designed to amplify the VJ region (and a relatively short part of Cα). High fidelity *Taq* polymerase was used for amplification of spleen cDNA from individual B4, and the resulting PCR-fragment was cloned. In total, 48 clones were analyzed. The 48 cDNA clones contained 25 different Jα gene segments (Table S1 in Supplementary Material). Out of the 48 clones, 45 were in frame. One clone had a stop codon (caused by point mutation) in the V sequence while two clones had frameshifts at the VJ junction. Alignment of the 48 cDNA clones indicated the presence of two subgroups, highly similar to B4-34 (differing at two nucleotide positions). The subgroups were treated separately before the total number of substitutions at each position was calculated. Substitutions at positions 82 and 95 were considered allotypic differences as they had two alternative nucleotides represented by approximately 50% each (Figure S2 in Supplementary Material).

Of the total 102 substitutions, transitions (53%) were more common than transversions (47%) with a T/V ratio of 1.12. Transversions were relatively abundant in FR2 and complementary determining region 2 (CDR2) with a T/V ratio of 0.3. When there is no bias toward transition or transversion, the theoretical ratio of transition to transversion is 0.5 for random substitutions. The overall ratio of replacement to synonymous substitutions was 2.5. Replacement substitutions were more frequent than silent mutations in both CDRs and FRs, except for FR4 where silent mutations were dominant (Table S7 in Supplementary Material). SHM studies in mammals and other teleost Ig variable genes have shown relatively high T/V and R/S mutation ratios (30).

### Mono, Di, and Trinucleotide Targets in the Vα1 Gene

The MIs for mononucleotides in the B4-34 group showed that nucleotides were preferentially targeted in the order: G > C > A~T. Of total mutations, G nucleotides were mutated 56.8%, followed by C (21.5%), and then A and T (10.8%). At G nucleotides 54% substitutions were transitions from G→A and transversions were G→T (41.4%) and G→C (10.4%) (Table 4). Analysis of dinucleotide MIs showed that CG and GC were the preferred targets for mutation, while AT were mutational cold spots (Table S8 in Supplementary Material). GC was found to be a significant target for mutation in human and catfish Ig heavy chain genes and zebrafish Ig light chain genes (28, 30, 47). When the analysis of the wrasse B4-34 group was expanded to trinucleotides, CG and GC dinucleotides were targeted most in GCG and CGA. Other combinations with significant MIs were AGG, GGT, and GAT (Table S9 in Supplementary Material).

**Table 4:** Substitutions and mutability index (MI) of T cell receptor Vα mononucleotides.

From	Substitutions				Observed mutations	Expected mutations	MI
	A	C	G	T			
A	–	2	9	0	11	28.5	0.38^a^
C	16	–	2	4	22	24.6	0.89
G	28	6	–	24	58	22.5	2.57^a^
T	0	11	0	–	11	26	0.42

*In total, 22,481 nucleotides were analyzed from B4-34 (A = 6342, T = 5746, G = 4961, C = 5432). There were 102 point mutations. MI values were calculated by dividing observed number of mutations to expected number of mutations. The observed and expected numbers of mutations were compared by χ^2^ analysis and significant differences are indicated on MI values*.

*^a^Statistically significant by χ^2^ test (*p* < 0.001)*.

## Discussion

The present study has shown that the TCRα genes in the teleost Ballan wrasse are subjected to SHM, and that this process also introduces some diversity in Cα. Similar to the situation in other teleosts (17, 23) the Cα gene in Ballan wrasse is polymorphic. Analysis of 37 fish identified 6 allotypes of Cα, each with 1–3 amino acid substitutions. Although TCR polymorphism is widespread among teleost fish it is tempting to suggest that some of the TCR Cα diversity observed in teleost cDNA pools might be a result of SHM.

The first attempt to amplify TCRα cDNA was based on a primer pair from a leader exon to the end of Cα, revealing four families of Vα genes. To analyze the SHM in a single Vα gene we selected the B4-34 as template and made the forward primer from the boundary of the leader and Vα exons. The resulting sequences were divided into two groups and treated separately to avoid over-estimation of mutation frequencies (Figure S2 in Supplementary Material). The two groups might represent two highly similar V genes which have been amplified by the B4-34 primer pair.

**Figure 5:**
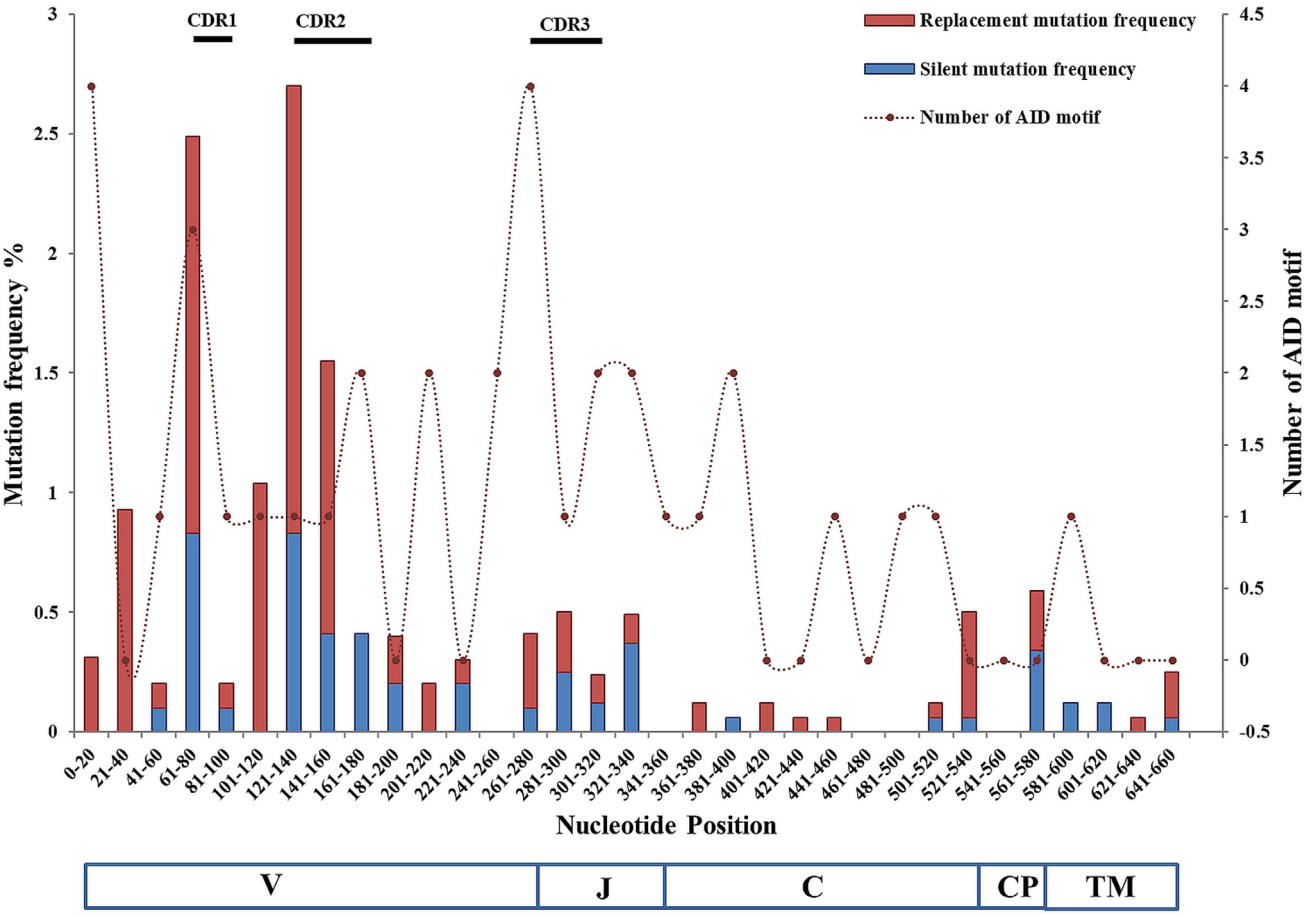
Distribution of mutation frequencies and number of activation-induced cytidine deaminase (AID) hotspot motifs in VJ and C regions of Ballan wrasse TCRα cDNA clones. The frequencies of silent and replacement mutations are plotted at the left vertical axis, while numbers of AID motifs are plotted at the right vertical axis; against nucleotide position intervals. The VJ region represents the combined mutation frequencies of 48 unique VJ cDNA clones, while the C region represents 79 unique cDNA clones. Numbers of AID hotspot motifs are represented as dashed lines. The diagram also indicates the location of V, J, and the constant Ig, connecting peptide, and TM regions. Primers included the first nine nucleotides of the V gene and the cytoplasmic region of Cα and were, therefore, not included in the mutability analysis. Position of the CDRs is depicted as bars over the graph.

The distribution of substitutions in TCR VJ and Cα is shown in Figure 5. The substitution rates were plotted against 20 bps nucleotide intervals. The first nucleotide corresponds to the third amino acid of FR1. Primers were designed from the start of the Vα gene and the first codons were, therefore, not included in the calculations. In the VJ region, CDR2 showed the highest frequency of substitutions. In Ballan wrasse TCRα the frequency of AID motifs was found to be much higher in the V/J genes (~8/100 bps) than in the Cα gene (4/100 bps), but no direct relationship was found between mutation frequencies and AID motifs in this study. In the VJ region, 6.8% of the mutations were present in AID motifs, while 9.2% were linked to WA/TW motifs, as compared to 40% in Cα. In a study of Ig variable genes it was found that SHM do occur in the absence of AID motifs and were predominantly G to C substitutions although the mutation frequency was lower than found in the presence of AID motifs (48). Mutability index analysis confirmed that nucleotides were targeted differently in the VJ and Cα region. In the VJ region, G nucleotides were targeted in CG and GC dinucleotides, and at GCG and GGG trinucleotide combinations. The mutations in Cα were primarily on A nucleotides, targeted mostly at AA (WA motif) and AT dinucleotides, indicating a mismatch repair mechanism employing polymerase η or other low fidelity polymerases (26). Replacement substitutions were dominant in both CDR1 and CDR2 and typically conservative. In a study of mouse TCR, extensive diversification by mutagenesis of CDR1 and CDR2 did not affect MHC binding (49), demonstrating that SHM of these regions is acceptable. In both Vα and Cα, replacement mutations were twice as frequent as silent mutations. Mutation frequencies in VJ versus Cα were found to be 4.5/1,000 bps and 1.76/1,000 bps, respectively. Key residues in Cα were conserved, showing that replacement substitutions had no impact on structural stability or interactions with CD3. Targeting of Cα is likely a side effect of SHM in the VJ region, like in the Ig light chain genes of zebrafish (30).

In the initial amplification and cloning of Ballan wrasse TCRα cDNAs about 24% of the clones had stop codons or were out of frame in the VJ region. The abundance of non-functional TCRα transcripts was similar in thymus and spleen. When narrowing the PCR-amplification to the B4-34 gene(s) the frequency of transcripts with stop codons or frame shifts decreased to 6.25%. Thus, it appears that there is a significant amount of non-functional transcripts in circulation, while some subpopulations of functional clones expand. In salmon, it was found that approximately 32% of TCRβ transcripts and 10% of TCRα transcripts in blood lymphocytes had stop codons or were out of frame (18, 50). In another study of salmon, about 10% of the TCRα transcripts from thymus were non-functional (20). Corresponding frequencies in rainbow trout thymus were 32% for TCRβ and 12.5% for TCRα (11, 51). In the amphibian Mexican axolotl more than 30% of the TCRβ transcripts from thymus and spleen, and 13.6% of TCRα transcripts from thymus were sterile (52). The high fraction of non-functional TCRβ transcripts in ectothermic animals contrasts the situation in mammals where these mRNAs are eliminated (53, 54), although not absolutely (55). A more “leaky” system in cold-blooded animals might be a result of less efficient cell proliferation and control mechanisms compared to higher vertebrates.

From the first amplification, TCRα clones which were in frame counted for 72% of the Cα mutations. As almost all replacement mutations were conservative there is no reason to believe that these are not incorporated into the functional T cell repertoire. Considering that we find somatic TCRα mutations in thymus as well as in spleen, SHM is probably involved in the diversification of the pre-immune TCR repertoire in Ballan wrasse, at the same time introducing some Cα diversity. A survey of translated TCRα ESTs in public databases revealed many amino acid substitutions in Atlantic salmon TCR Cα as well (Figure S3 in Supplementary Material). Thus, it is plausible to assume that SHM of TCR is a common phenomenon in teleost fish. However, SHM in mature T cells of wrasse cannot be ruled out either, and is an interesting topic for further research. SHM of TCR is generally believed to be restricted. Typically, an increase in TCR affinity after secondary challenge is minor compared to the affinity maturation of antibodies in mammals (56). It has been suggested that high-affinity TCR clones might be unfavorable due to prolonged binding, impairing serial interactions. On the other hand, somatic mutation of TCR genes in mature T cells has been documented (31, 32), suggesting that SHM of TCRs can occur under certain conditions, e.g., chronic activation (33). Vaccination of fish might facilitate conditions that trigger inappropriate immune responses (57, 58), but wild caught fish were used in the present analysis, implying that the results presented here are a normal situation in adult fish.

In conclusion, this study has shown that the TCRα in the teleost Ballan wrasse is subjected to SHM. The mutation frequency was highest in CDR2, although mutations were also evident in the constant part and FRs of TCRα. A high-throughput sequencing approach will be an interesting future study that can provide a more complete overview of the effects of SHM during the development of the TCR repertoire in the teleost fish.

## Ethics Statement

All work complied with relevant ethical guidelines and regulations. Ethical approval was not required as the study did not involve transport or experiments on live fish.

## Author Contributions

SB and IH designed the study. SB did experimental work and data analysis. KL and ØS worked on high-throughput sequence data. IH took part in data analysis and supervised the whole study.

## Conflict of Interest Statement

The authors declare that the research was conducted in the absence of any commercial or financial relationships that could be construed as a potential conflict of interest. The reviewer KM and handling Editor declared their shared affiliation.

## Footnotes

^1^https://github.com/najoshi/sickle.

^2^http://www.bioinformatics.babraham.ac.uk/projects/fastqc/.

^3^http://blast.ncbi.nlm.nih.gov/.

^4^http://www.expasy.org/.

^5^http://www.ebi.ac.uk/.
