# Supplementary material for: T Cell Receptor Alpha Chain Genes in the Teleost Ballan Wrasse (Labrus bergylta) Are Subjected to Somatic Hypermutation
Source: Front Immunol. 2018 May 22;9:1101. doi: 10.3389/fimmu.2018.01101 (PMC5972329; doi:10.3389/fimmu.2018.01101)
Supplement: Supplementary file 1 [file table_1.docx]

**Supplementary TABLE 1. Position of gene segments in the TCRα/δ locus (LaB_20160104_scaffold_928)**

| **Number** | **J segments found in 79 cDNA clones** | **J segments found in the B4-34 group** | **Position on scaffold 928** | |
| --- | --- | --- | --- | --- |
| Cα Exon 3 |  |  | 5048 | 5245 |
| Cα Exon 2 |  |  | 4891 | 4932 |
| Cα Exon 1 |  |  | 4698 | 4795 |
|  |  |  |  |  |
| Jα1 |  | 49 | 5719 | 5778 |
| Jα2 |  |  | 6820 | 6879 |
| Jα3 |  |  | 7687 | 7779 |
| Jα4 |  |  | 8258 | 8323 |
| Jα5 | B6_23 | 53 | 9108 | 9164 |
| Jα6 |  | 24 | 9761 | 9818 |
| Jα7 | B6_20, B1_49 |  | 10689 | 10742 |
| Jα8 |  | 23 | 10916 | 10978 |
| Jα9 | B6_9, B1_22 | 10, 39, 52 | 11381 | 11443 |
| Jα10 |  | 1 | 12048 | 12110 |
| Jα11 |  |  | 12727 | 12789 |
| Jα12 | B4_27 |  | 13342 | 13504 |
| Jα13 | B6_26 | 4 | 13941 | 14003 |
| Jα14 |  |  | 14848 | 14913 |
| Jα15 | B1_28, B4_49 |  | 15595 | 15660 |
| Jα16 | B1_47 |  | 16263 | 16328 |
| Jα17 | B4_50 |  | 16959 | 17021 |
| Jα18 | B1_20 |  | 17258 | 17320 |
| Jα19 | B4_42 |  | 17807 | 17869 |
| Jα20 |  |  | 18519 | 18607 |
| Jα21 | B1_13 |  | 19492 | 19550 |
| Jα22 | B4_17, B4_34 | 29 | 20228 | 20290 |
| Jα23 | B6_30 |  | 20574 | 20639 |
| Jα24 |  |  | 21061 | 21120 |
| Jα25 | B6_12, B6_34, B6­_45, B4_46, B4_54 | 8, 9 | 21787 | 21849 |
| Jα26 | B6_13, B6_18, B6_4, B6_21, B6_50, B4_19 |  | 22847 | 22912 |
| Jα27 |  | 6 | 23661 | 23723 |
| Jα28 | B1_17, B1_35, B1_52 |  | 24117 | 24189 |
| Jα29 | B6_15 |  | 24925 | 24987 |
| Jα30 |  |  | 25383 | 25336 |
| Jα31 |  | 42 | 26177 | 26239 |
| Jα32 | B1_29, B4_32 |  | 26636 | 26708 |
| Jα33 | B6_17, B4_33 | 5 | 27453 | 27515 |
| Jα34 | B4_29 |  | 27959 | 28016 |
| Jα35 | B6_40 | 22 | 28429 | 28486 |
| Jα36 | B1_37, B4_39, B4_47 |  | 29452 | 29514 |
| Jα37 | B6_2, B6_5 | 46 | 29765 | 29821 |
| Jα38 |  |  | 30224 | 30289 |
| Jα39 | B6_38, B6_39, B1_24, B4_25 |  | 31203 | 31259 |
| Jα40 |  |  | 31921 | 31977 |
| Jα41 |  |  | 32732 | 32797 |
| Jα42 | B6_16, B6_39 |  | 33727 | 33783 |
| Jα43 | B6_51 | 35 | 34441 | 34497 |
| Jα44 |  |  | 35168 | 35233 |
| Jα45 | B1_32 |  | 35850 | 35909 |
| Jα46 | B1_9 | 41, 50 | 36598 | 36660 |
| Jα47 | B1_18 | 47 | 37032 | 37091 |
| Jα48 |  |  | 37362 | 37421 |
| Jα49 |  |  | 37674 | 37739 |
| Jα50 |  | 25 | 38017 | 38076 |
| Jα51 |  |  | 38336 | 38401 |
| Jα52 | B6_14 |  | 38671 | 38729 |
| Jα53 |  | 31 | 38989 | 39054 |
| Jα54 | B4_48 |  | 39324 | 39383 |
| Jα55 |  | 12, 13, 33 | 40461 | 40523 |
| Jα56 |  | 43 | 41038 | 41097 |
| Jα57 |  |  | 41807 | 41867 |
| Jα58 |  |  | 42052 | 42111 |
| Jα59 | B4_18, B4_40, B4_44 | 27, 36, 38,44, 48, 51 |  |  |
| Jα60 | B1_2 |  | 43289 | 43351 |
| Jα61 |  |  | 43807 | 43869 |
| Jα62 | B4_20, B4_45 | 7, 14 | 44180 | 44242 |
| Jα63 | B6_36, B6_11 | 2, 11, 16, 26 | 44541 | 44626 |
| Jα64 |  | 30, 32, 37, 40 | 44953 | 45016 |
| Jα65 |  |  | 45332 | 45394 |
|  |  |  |  |  |
| Cδ Exon 3 |  |  | 57680 | 57727 |
| Cδ Exon 2 |  |  | 58000 | 57974 |
| Cδ Exon 1 |  |  | 59290 | 59605 |
|  |  |  |  |  |
| Jδ1 |  |  | 63621 | 63668 |
|  |  |  |  |  |
| Dδ1 |  |  | 64048 | 64076 |
| Dδ2 |  |  | 65060 | 65077 |
|  |  |  |  |  |
| SMG-7 |  |  | 92446 | 94195 |
