# Supplementary material for: T Cell Receptor Alpha Chain Genes in the Teleost Ballan Wrasse (Labrus bergylta) Are Subjected to Somatic Hypermutation
Source: Front Immunol. 2018 May 22;9:1101. doi: 10.3389/fimmu.2018.01101 (PMC5972329; doi:10.3389/fimmu.2018.01101)
Supplement: Supplementary file 2 [file table_2.docx]

**Supplementary TABLE 2. Position of gene segments in the TCRα/δ locus (LaB_20160104_scaffold_4467)**

| **Gene** | **Position on scaffold 4467** | |
| --- | --- | --- |
| Cα Exon 1 | 9208 | 9405 |
| Cα Exon 2 | 9521 | 9562 |
| Cα Exon 3 | 9656 | 9761 |
|  |  |  |
| Jα1 | 8643 | 8682 |
| Jα2 | 7573 | 7632 |
| Jα3 | 6703 | 6765 |
| Jα4 | 6096 | 6161 |
| Jα5 | 5263 | 5319 |
| Jα6 | 4606 | 4665 |
| Jα7 | 3669 | 3731 |
| Jα8 | 3026 | 3076 |
| Jα9 | 2560 | 2622 |
| Jα10 | 1894 | 1956 |
| Jα11 | 1215 | 1277 |
| Jα12 | 500 | 562 |
| Jα13 | 2 | 63 |
