# Supplementary material for: T Cell Receptor Alpha Chain Genes in the Teleost Ballan Wrasse (Labrus bergylta) Are Subjected to Somatic Hypermutation
Source: Front Immunol. 2018 May 22;9:1101. doi: 10.3389/fimmu.2018.01101 (PMC5972329; doi:10.3389/fimmu.2018.01101)
Supplement: Supplementary file 3 [file table_3.docx]

**Supplementary TABLE 3. Distribution of TCR Cα alleles in 37 individuals of Ballan wrasse**

| **Number of individual** | **Name of individual** | **Alleles** | | | | | |
| --- | --- | --- | --- | --- | --- | --- | --- |
|  |  | **A** | **B** | **C** | **D** | **E** | **F** |
| GenBank | 928/4467 | x | x |  |  |  |  |
| 1 | T1 |  |  |  | x |  | x |
| 2 | T2 |  | xx |  |  |  |  |
| 3 | T3 |  |  | x | x |  |  |
| 4 | T4 | x | x |  |  |  |  |
| 5 | T5 |  |  |  | x | x |  |
| 6 | T6 |  |  | x | x |  |  |
| 7 | 182-2 |  | xx |  |  |  |  |
| 8 | 182-3 |  | xx |  |  |  |  |
| 9 | 182-4 |  | xx |  |  |  |  |
| 10 | 182-5 |  | x | x |  |  |  |
| 11 | 182-6 |  |  | x | x |  |  |
| 12 | 182-8 | xx |  |  |  |  |  |
| 13 | 182-9 |  | xx |  |  |  |  |
| 14 | 182-10 | x |  | x |  |  |  |
| 15 | 182-11 |  | x | x |  |  |  |
| 16 | 182-12 |  |  | xx |  |  |  |
| 17 | 182-13 | x |  | x |  |  |  |
| 18 | 182-14 |  | x |  | x |  |  |
| 19 | 182-15 | x | x |  |  |  |  |
| 20 | 182-16 | x | x |  |  |  |  |
| 21 | 182-17 |  | xx |  |  |  |  |
| 22 | 182-18 |  | x |  | x |  |  |
| 23 | 182-19 | xx |  |  |  |  |  |
| 24 | 182-21 | x |  | x |  |  |  |
| 25 | 182-22 |  | xx |  |  |  |  |
| 26 | 182-23 |  | x |  | x |  |  |
| 27 | 182-24 | x |  | x |  |  |  |
| 28 | 182-37 |  | x |  | x |  |  |
| 29 | 182-38 |  | x | x |  |  |  |
| 30 | 182-39 |  | x |  | x |  |  |
| 31 | 182-41 | x | x |  |  |  |  |
| 32 | 182-42 | x |  |  | x |  |  |
| 33 | 182-43 |  | xx |  |  |  |  |
| 34 | 182-46 |  | x | x |  |  |  |
| 35 | B1 | x | x |  |  |  |  |
| 36 | B4 |  | xx |  |  |  |  |
| 37 | B6 |  | x |  |  |  | x |
