# Supplementary material for: T Cell Receptor Alpha Chain Genes in the Teleost Ballan Wrasse (Labrus bergylta) Are Subjected to Somatic Hypermutation
Source: Front Immunol. 2018 May 22;9:1101. doi: 10.3389/fimmu.2018.01101 (PMC5972329; doi:10.3389/fimmu.2018.01101)
Supplement: Supplementary file 4 [file table_4.docx]

**Supplementary TABLE 4. Mutations in Ballan wrasse TCRα cDNA clones**

| **# of clones** | **Clone** | **Nucleotide** | | **Mutation type** | | **Position**  **aa** | **Motif** |
| --- | --- | --- | --- | --- | --- | --- | --- |
| **FR1 (3-26)** | | | | | | | |
| 3 | 19, 24, 47 | G→A | T**G**C→T**A**C | A→T | T | 8 |  |
| 1 | 47 | T→C | G**T**G→G**C**G | V→A | T | 10 |  |
| 1 | 29 | C→T | **C**TG→**T**TG | Silent L | T | 21 |  |
| 1 | 33 | T→C | **T**CC→**C**CC | S→P | T | 22 |  |
| **CDR1 (27-38)** | | | | | | | |
| 16 | 6, 8, 9, 12, 13, 18, 22, 33, 37, 39, 41, 42, 43, 47, 52, 53 | G→A | **G**AT→**A**AT | D→N | T | 37 |  |
| **FR2 (29-55)** | | | | | | | |
| 1 | 24 | A→G | CA**A**→CA**G** | Silent Q | T | 43 |  |
| 1 | 6 | C→T | **C**AG→**T**AG | Stop Codon | T | 44 |  |
| 10 | 6, 19, 20, 24, 37, 39, 43, 46, 50, 52 | G→T | **G**AT→**T**AT | D→Y | V | 45 |  |
| 1 | 9 | T→C | T**T**C→T**C**C | F→S | T | 52 | AID |
| **CDR2 (55-65)** | | | | | | | |
| 8 | 12, 13, 14, 19, 24, 33, 44, 47 | C→A | AT**C**→AT**A** | Silent I | V | 56 |  |
| 11 | 12, 13, 14, 18,19, 24, 31, 33, 44, 47, 50 | G→T | A**G**C→A**T**C | S→I | V | 57 |  |
| 6 | 5, 22, 32, 40, 41, 53 | G→C | A**G**C→A**C**A | S→T | V | 57 |  |
| 6 | 5, 22, 32, 40, 41, 53 | C→A | **A**G**C**→**A**C**A** | S→T | V | 57 |  |
| 3 | 11, 17, 26 | G→A | **G**AC→**A**AC | D→N | T | 58 |  |
| 2 | 14, 44 | C→A | A**C**T→A**A**T | T→N | V | 59 |  |
| 1 | 27 | A→G | TC**A**→TC**G** | Silent S | T | 60 |  |
| 1 | 53 | A→C | CC**A**→CC**C** | Silent P | V | 62 | WA/  AID |
| 2 | 14, 44 | T→C | AC**T**→AC**C** | Silent T | T | 63 | WA / AID |
| 4 | 12, 13, 24, 33 | G→A | GT**G**→GT**A** | Silent V | T | 65 |  |
| **FR3 (66-104)** | | | | | | | |
| 1 | 12 | A→G | C**A**C→C**G**C | H→R | T | 71 |  |
| 1 | 30 | T→C | CC**T**→CC**C** | Silent P | T | 74 |  |
| 1 | 51 | C→T | **C**TG→**T**TG | Silent L | T | 76 |  |
| 1 | 50 | A→G | **A**AA→**G**AA | K→E | T | 79 | WA |
| 1 | 21 | A→G | G**A**G→G**G**G | E→G | T | 82 |  |
| 1 | 23 | C→T | **C**GT→**T**GT | R→C | T | 86 | AID |
| 2 | 19, 47 | G→T | GT**G**→GT**T** | Silent V | T | 87 |  |
| 1 | 41 | A→G | **A**TC→**G**TC | I→V | T | 91 |  |
| 1 | 46 | T→C | **T**AC→**C**AC | Y→H | T | 103 | WA/  AID |
| 1 | 43 | T→C | TG**T**→TG**C** | Silent C | T | 104 | AID |
| **CDR3 (105-117)** | | | | | | | |
| 2 | 25, 42 | C→G | **C**TG→**G**TG | L→V | V | 106 |  |
| 1 | 51 | G→T | AAG→AAT | Silent N | V | 107 |  |
| 1 | 24 | A→C | **A**AC→ **C**AC | N→H | V | 107 | WA |
| 1 | 29 | A→G | A**A**T→A**G**T | N→S | T | 108 | WA |
| 1 | 40 | T→C | CCT→CCC | Silent P | T | 108 | WA |
| 1 | 31 | G→A | **G**GT→**A**GT | G→S | T | 113 |  |
| **FR4 (118-128)** | | | | | | | |
| 1 | 29 | G→A | AA**G**→AA**A** | Silent K | T | 115 |  |
| 1 | 51 | A→G | AAA→AAG | Silent K | T | 123 | WA |
| 1 | 25 | T→C | A**T**T→A**C**T | I →T | T | 126 | TW |
| 1 | 04 | T→C | TTT→TTC | Silent F | T | 125 | TW |
| 1 | 49 | A→G | AGG→CGG | Silent R | T | 127 |  |
|  |  |  |  |  |  |  |  |
| **Cα** | | | | | | | |
| 1 | B1­-9 | A→G | C**A**C→C**G**C | H→R | T | 8 | - |
| 1 | B1­-17 | A→T | A**A**A→A**T**A | K→I | V | 9 | WA/  AID |
| 2 | B6-35, B6-40 | A→G | AA**A**→AA**G** | Silent K | T | 9 | WA  /AID |
| 1 | B6-61 | G→A | CT**G**→CT**A** | Silent L | T | 10 | - |
| 1 | B6-38 | G→A | AC**G**→AC**A** | Silent T | T | 16 | - |
| 1 | B4-18 | A→G | **A**CC→**G**GC | T→A | T | 24 | - |
| 1 | B6-14 | A→C | C**A**A→C**C**A | Q→P | V | 25 |  |
| 1 | B6-45 | G→A | **G**TG→**A**TG | V→M | T | 29 | - |
| 1 | B1­-24 | T→C | C**T**T→C**C**T | L→P | T | 33 | TW |
| 1 | B4-17 | A→G | AC**A**→AC**G** | Silent T | T | 55 | WA |
| 1 | B4-18 | A→G | G**A**A→G**G**A | E→G | T | 57 | WA |
| 1 | B6-25 | A→G | AA**A**→AA**G** | Silent K | T | 60 | WA |
| 2 | B1-2, B1-37 | add |  | Frameshift |  | 60 | - |
| 1 | B4-44 | A→G | C**A**A→C**G**A | Q→R | T | 61 | WA |
| 3 | B1­-28, B4-40, B4-43 | A→G | A**A**A→A**G**A | K→R | T | 62 | WA |
| 2 | B1­-22, B4-32 | A→G | G**A**T→G**G**T | D→G | T | 64 | - |
| 1 | B4-20 | A→G | G**A**A→G**G**A | W→G | T | 65 | WA |
| 1 | B4-25 | A→G | GG**A**→GG**G** | Silent G | T | 74 | - |
| 1 | B6-40 | T→C | GA**T**→GA**C** | Silent D | T | 75 | - |
| 4 | B1­-29, B4-54, B6-2, B6-4 | A→G | A**A**T→A**G**T | N→S | T | 78 | WA |
| 3 | B4-22, B6-22,  B6-51 | A→G | CG**A**→CG**G** | Silent R | T | 79 | - |
| 1 | B4-48 | A→G | **A**TG→**G**TG | M→V | T | 82 | - |
| 1 | B6-51 | T→C | G**T**C→G**C**C | V→A | T | 83 | - |
| 1 | B4-25 | T→C | C**T**G→C**C**G | L→P | T | 85 | - |
| 1 | B1­-22 | T→C | TC**T**→TC**C** | Silent S | T | 87 | - |
| 1 | B1­-13 | T→C | GT**T**→GT**C** | Silent V | T | 91 | TW |
| 1 | B6-28 | add |  | Frameshift |  | 91 | - |
| 1 | B6-30 | add |  | Frameshift |  | 92 | - |
| 1 | B6-58 | T→C | C**T**C→C**C**C | L→P | T | 93 | - |
| 1 | B6-16 | G→C | AA**G**→AA**C** | K→N | V | 99 | - |
| 1 | B4-33 | A→G | **A**CC→**G**CC | T→A | T | 100 | - |
| 1 | B4-19 | C→T | AC**C→**AC**T** | Silent T | T | 100 | - |
| 1 | B4-47 | A→G | **A**TC→**G**TC | I→V | T | 101 | - |
| 1 | B6-18 | T→C | G**T**C→G**C**C | V→A | T | 105 | - |
