# Supplementary material for: T Cell Receptor Alpha Chain Genes in the Teleost Ballan Wrasse (Labrus bergylta) Are Subjected to Somatic Hypermutation
Source: Front Immunol. 2018 May 22;9:1101. doi: 10.3389/fimmu.2018.01101 (PMC5972329; doi:10.3389/fimmu.2018.01101)
Supplement: Supplementary file 5 [file table_5.docx]

**Supplementary TABLE 5.** **Mutability index of TCR Cα dinucleotides**

| **Dinucleotide** | **Number of times sequence appear**  **in all clones** | **Expected**  **mutations** | **Observed**  **mutations** | **Mutability index** |
| --- | --- | --- | --- | --- |
| **AA** | 1621 | 3.05 | 13 | 4.26 ^a^ |
| **AG** | 1156 | 2.17 | 6 | 2.75 ^b^ |
| **AC** | 2052 | 3.86 | 4 | 1.03 |
| **AT** | 1178 | 2.21 | 8 | 3.60 ^a^ |
| **GA** | 2084 | 3.92 | 11 | 2.80 ^a^ |
| **GG** | 1456 | 2.74 | 0 | 0 |
| **GC** | 1149 | 2.16 | 1 | 0.46 |
| **GT** | 1269 | 2.38 | 3 | 1.2 |
| **CA** | 1978 | 3.72 | 5 | 1.34 |
| **CG** | 1691 | 3.18 | 1 | 0.31 |
| **CC** | 1347 | 2.53 | 1 | 0.39 |
| **CT** | 1578 | 2.97 | 3 | 1 |
| **TA** | 319 | 0.60 | 0 | 0 |
| **TG** | 1815 | 3.41 | 2 | 0.58 |
| **TC** | 1893 | 3.56 | 4 | 1.12 |
| **TT** | 788 | 1.48 | 2 | 1.34 |

The observed and expected numbers of mutations were compared by χ^2^ analysis and significant differences are indicated on mutability index values.

**^a^** statistically significant by χ^2^ test (*p* < 0.001)

**^b^** statistically significant by χ^2^ test (*p* < 0.01)
