# Supplementary material for: T Cell Receptor Alpha Chain Genes in the Teleost Ballan Wrasse (Labrus bergylta) Are Subjected to Somatic Hypermutation
Source: Front Immunol. 2018 May 22;9:1101. doi: 10.3389/fimmu.2018.01101 (PMC5972329; doi:10.3389/fimmu.2018.01101)
Supplement: Supplementary file 6 [file table_6.docx]

**Supplementary TABLE 6. Mutability index of TCR Cα trinucleotides**

| **Trinucleotide** | **Number of times**  **sequence appear**  **in all clones** | **Expected**  **mutations** | **Observed**  **mutations** | **Mutability**  **index** |
| --- | --- | --- | --- | --- |
| **AAA** | 481 | 0.87 | 11 | 12.58 ^a^ |
| **AGA** | 444 | 0.80 | 6 | 7.43 ^a^ |
| **ACA** | 479 | 0.87 | 4 | 4.59 ^a^ |
| **ATA** | 80 | 0.14 | 0 | 0 |
| **AAT** | 309 | 0.56 | 8 | 14.25 ^a^ |
| **AGT** | 82 | 0.14 | 1 | 6.71 ^c^ |
| **ACT** | 356 | 0.64 | 3 | 4.63 ^b^ |
| **ATT** | 158 | 0.28 | 0 | 0 |
| **AAC** | 750 | 1.36 | 5 | 3.66 ^b^ |
| **AGC** | 130 | 0.23 | 0 | 0 |
| **ACC** | 630 | 1.14 | 3 | 2.62 |
| **ATC** | 626 | 1.13 | 6 | 5.27 ^a^ |
| **AAG** | 398 | 0.72 | 3 | 4.14 ^b^ |
| **AGG** | 502 | 0.91 | 0 | 0 |
| **ACG** | 591 | 1.07 | 0 | 0 |
| **ATG** | 314 | 0.57 | 3 | 5.25 ^b^ |
| **GAG** | 432 | 0.78 | 4 | 5.09 ^a^ |
| **GGG** | 241 | 0.43 | 0 | 0 |
| **GCG** | 237 | 0.43 | 0 | 0 |
| **GTG** | 477 | 0.86 | 1 | 1.15 |
| **GAT** | 553 | 1.00 | 4 | 3.98 ^b^ |
| **GGT** | 318 | 0.57 | 1 | 1.73 |
| **GCT** | 276 | 0.50 | 0 | 0 |
| **GTT** | 157 | 0.28 | 1 | 3.50 |
| **GAA** | 548 | 0.99 | 6 | 6.02 ^a^ |
| **GGA** | 479 | 0.87 | 1 | 1.14 |
| **GCA** | 238 | 0.43 | 1 | 2.31 |
| **GTA** | 79 | 0.14 | 0 | 0 |
| **GAC** | 631 | 1.14 | 2 | 1.74 |
| **GGC** | 658 | 1.19 | 0 | 0 |
| **GCC** | 477 | 0.86 | 0 | 0 |
| **GTC** | 558 | 1.01 | 2 | 1.97 |
| **CAC** | 584 | 1.06 | 2 | 1.88 |
| **CGC** | 162 | 0.29 | 0 | 0 |
| **CCC** | 241 | 0.43 | 0 | 0 |
| **CTC** | 393 | 0.71 | 1 | 1.40 |
| **CAG** | 328 | 0.59 | 0 | 0 |
| **CGG** | 476 | 0.86 | 1 | 1.15 |
| **CCG** | 545 | 0.99 | 0 | 0 |
| **CTG** | 709 | 1.28 | 2 | 1.55 |
| **CAA** | 830 | 1.50 | 4 | 2.65 ^c^ |
| **CGA** | 536 | 0.97 | 4 | 4.10 ^b^ |
| **CCA** | 405 | 0.73 | 4 | 5.43 ^a^ |
| **CTA** | 81 | 0.14 | 0 | 0 |
| **CAT** | 236 | 0.42 | 2 | 4.66 ^c^ |
| **CGT** | 474 | 0.86 | 1 | 1.16 |
| **CCT** | 395 | 0.71 | 1 | 1.39 |
| **CTT** | 395 | 0.71 | 1 | 1.39 |
| **TAT** | 80 | 0.14 | 0 | 0 |
| **TGT** | 395 | 0.71 | 0 | 0 |
| **TCT** | 473 | 0.85 | 1 | 1.16 |
| **TTT** | 160 | 0.29 | 0 | 0 |
| **TAG** | 0 | 0 | 0 | - |
| **TGG** | 398 | 0.72 | 0 | 0 |
| **TCG** | 318 | 0.57 | 0 | 0 |
| **TTG** | 315 | 0.57 | 0 | 0 |
| **TAC** | 160 | 0.29 | 0 | 0 |
| **TGC** | 236 | 0.42 | 1 | 2.33 |
| **TCC** | 237 | 0.43 | 2 | 4.64 ^c^ |
| **TTC** | 316 | 0.57 | 0 | 0 |
| **TAA** | 79 | 0.14 | 0 | 0 |
| **TGA** | 706 | 1.28 | 5 | 3.89 ^b^ |
| **TCA** | 787 | 1.42 | 2 | 1.39 |
| **TTA** | 79 | 0.14 | 0 | 0 |

The observed and expected numbers of mutations were compared by χ^2^ analysis and significant differences are indicated on mutability index values.

**^a^** statistically significant by χ^2^ test (*p* < 0.001)

**^b^** statistically significant by χ^2^ test (*p* < 0.01)

**^c^** statistically significant by χ^2^ test (*p* < 0.05)
