# Supplementary material for: T Cell Receptor Alpha Chain Genes in the Teleost Ballan Wrasse (Labrus bergylta) Are Subjected to Somatic Hypermutation
Source: Front Immunol. 2018 May 22;9:1101. doi: 10.3389/fimmu.2018.01101 (PMC5972329; doi:10.3389/fimmu.2018.01101)
Supplement: Supplementary file 7 [file table_7.docx]

**Supplementary TABLE 7. Number and ratio of replacement to silent and transition to transversion mutations in different regions of TCR VJ genes in Ballan wrasse**

|  | **Total mutations** | **Replace-**  **ment** | **Silent** | **R/S** | **Trans-**  **itions** | **Trans- versions** | **T/V** |
| --- | --- | --- | --- | --- | --- | --- | --- |
| **FR1**  **(1-26)** | 6 | 5 | 1 | 5 | 6 | 0 | - |
| **CDR1 (27-38)** | 16 | 16 | 0 | - | 16 | 0 | - |
| **FR2**  **(29-55)** | 13 | 12 | 1 | 12 | 3 | 10 | 0.3 |
| **CDR2 (56-65)** | 44 | 28 | 16 | 1.75 | 10 | 34 | 0.29 |
| **FR3**  **(66-104)** | 11 | 6 | 5 | 1.2 | 11 | 0 | - |
| **CDR3 (105-117)** | 7 | 5 | 2 | 2.5 | 3 | 4 | 0.75 |
| **FR4**  **(118-128)** | 5 | 1 | 4 | 0.25 | 5 | 0 | - |
| **Total** | **102** | **73** | **29** | **2.5** | **54** | **48** | **1.12** |
