# Supplementary material for: T Cell Receptor Alpha Chain Genes in the Teleost Ballan Wrasse (Labrus bergylta) Are Subjected to Somatic Hypermutation
Source: Front Immunol. 2018 May 22;9:1101. doi: 10.3389/fimmu.2018.01101 (PMC5972329; doi:10.3389/fimmu.2018.01101)
Supplement: Supplementary file 8 [file table_8.docx]

**Supplementary TABLE 8.**  **Mutability index of TCR Vα dinucleotides**

| **Dinucleotide** | **Number of times sequence appear**  **in all clones** | **Expected**  **mutations** | **Observed**  **mutations** | **Mutability index** |
| --- | --- | --- | --- | --- |
| **AA** | 834 | 6.24 | 5 | 0.80 |
| **AG** | 1162 | 8.7 | 12 | 1.37 |
| **AC** | 1292 | 9.6 | 6 | 0.62 |
| **AT** | 816 | 6.1 | 1 | 0.16 ^c^ |
| **GA** | 1191 | 8.9 | 19 | 2.1 ^a^ |
| **GG** | 483 | 3.6 | 10 | 2.76 ^a^ |
| **GC** | 600 | 4.49 | 21 | 4.67 ^a^ |
| **GT** | 1097 | 8.2 | 3 | 0.36 |
| **CA** | 1278 | 9.5 | 12 | 1.25 |
| **CG** | 244 | 1.8 | 10 | 5.4 ^a^ |
| **CC** | 756 | 5.6 | 1 | 0.17 |
| **CT** | 1828 | 13.6 | 8 | 0.58 |
| **TA** | 774 | 5.79 | 3 | 0.51 |
| **TG** | 1498 | 11.2 | 28 | 2.49 ^a^ |
| **TC** | 1448 | 10.84 | 12 | 1.10 |
| **TT** | 592 | 4.43 | 1 | 0.22 |

Mutability index values were calculated by dividing observed number of mutations to expected number of mutations. The observed and expected numbers of mutations were compared by χ^2^ analysis and significant differences are indicated on mutability index values.

**^a^** statistically significant by χ^2^ test (*p* < 0.001)

**^c^** statistically significant by χ^2^ test (*p* < 0.05)
