# Supplementary material for: T Cell Receptor Alpha Chain Genes in the Teleost Ballan Wrasse (Labrus bergylta) Are Subjected to Somatic Hypermutation
Source: Front Immunol. 2018 May 22;9:1101. doi: 10.3389/fimmu.2018.01101 (PMC5972329; doi:10.3389/fimmu.2018.01101)
Supplement: Supplementary file 9 [file table_9.docx]

**Supplementary TABLE 9. Mutability index of TCR Vα trinucleotides**

| **Trinucleotide** | **Number of times**  **sequence appear**  **in all clones** | **Expected**  **mutations** | **Observed**  **mutations** | **Mutability**  **index** |
| --- | --- | --- | --- | --- |
| **AAA** | 316 | 2.35 | 3 | 1.27 |
| **AGA** | 242 | 1.80 | 5 | 2.77 ^c^ |
| **ACA** | 337 | 2.51 | 3 | 1.19 |
| **ATA** | 160 | 1.19 | 1 | 0.84 |
| **AAT** | 228 | 1.70 | 1 | 0.58 |
| **AGT** | 394 | 2.94 | 1 | 0.33 |
| **ACT** | 700 | 5.22 | 7 | 1.34 |
| **ATT** | 121 | 0.90 | 0 | 0 |
| **AAC** | 363 | 2.70 | 4 | 1.48 |
| **AGC** | 261 | 1.94 | 17 | 8.72 ^a^ |
| **ACC** | 223 | 1.66 | 1 | 0.60 |
| **ATC** | 491 | 3.66 | 9 | 2.45 ^b^ |
| **AAG** | 172 | 1.28 | 2 | 1.56 |
| **AGG** | 202 | 1.50 | 19 | 12.6 ^a^ |
| **ACG** | 30 | 0.22 | 0 | 0 |
| **ATG** | 42 | 0.31 | 0 | 0 |
| **GAG** | 175 | 1.30 | 1 | 0.76 |
| **GGG** | 98 | 0.73 | 0 | 0 |
| **GCG** | 41 | 0.30 | 20 | 65.3 ^a^ |
| **GTG** | 401 | 2.99 | 10 | 3.34 ^a^ |
| **GAT** | 358 | 2.67 | 28 | 10.48 ^a^ |
| **GGT** | 105 | 0.78 | 9 | 11.53 ^a^ |
| **GCT** | 311 | 2.32 | 3 | 1.29 |
| **GTT** | 285 | 2.12 | 1 | 0.46 |
| **GAA** | 259 | 1.93 | 3 | 0 |
| **GGA** | 306 | 2.28 | 12 | 5.25 ^a^ |
| **GCA** | 173 | 1.29 | 0 | 0 |
| **GTA** | 226 | 1.68 | 0 | 0 |
| **GAC** | 339 | 2.53 | 8 | 3.16 ^a^ |
| **GGC** | 47 | 0.35 | 1 | 2.85 |
| **GCC** | 73 | 0.54 | 0 | 0 |
| **GTC** | 116 | 0.86 | 1 | 1.15 |
| **CAC** | 212 | 1.58 | 2 | 1.26 |
| **CGC** | 5 | 0.03 | 0 | 0 |
| **CCC** | 63 | 0.47 | 0 | 0 |
| **CTC** | 579 | 4.32 | 12 | 2.77 ^a^ |
| **CAG** | 703 | 5.24 | 19 | 3.62 ^a^ |
| **CGG** | 55 | 0.41 | 0 | 0 |
| **CCG** | 88 | 0.65 | 1 | 1.52 |
| **CTG** | 950 | 7.09 | 22 | 3.10 ^a^ |
| **CAA** | 213 | 1.59 | 2 | 1.25 |
| **CGA** | 109 | 0.81 | 9 | 11.06 ^a^ |
| **CCA** | 263 | 1.96 | 1 | 0.50 |
| **CTA** | 149 | 1.11 | 2 | 1.80 |
| **CAT** | 79 | 0.58 | 0 | 0 |
| **CGT** | 74 | 0.55 | 1 | 1.81 |
| **CCT** | 382 | 2.85 | 2 | 0.70 |
| **CTT** | 128 | 0.95 | 2 | 2.09 |
| **TAT** | 100 | 0.74 | 0 | 0 |
| **TGT** | 474 | 3.53 | 5 | 1.41 |
| **TCT** | 435 | 3.24 | 1 | 0.30 |
| **TTT** | 97 | 0.72 | 0 | 0 |
| **TAG** | 55 | 0.41 | 0 | 0 |
| **TGG** | 212 | 1.58 | 3 | 1.89 |
| **TCG** | 85 | 0.63 | 1 | 1.57 |
| **TTG** | 58 | 0.43 | 0 | 0 |
| **TAC** | 347 | 2.59 | 9 | 3.47 ^a^ |
| **TGC** | 286 | 2.13 | 4 | 1.87 |
| **TCC** | 459 | 3.42 | 2 | 0.58 |
| **TTC** | 241 | 1.79 | 1 | 0.55 |
| **TAA** | 272 | 2.03 | 2 | 0.98 |
| **TGA** | 474 | 3.53 | 21 | 5.94 ^a^ |
| **TCA** | 464 | 3.46 | 9 | 2.59 ^b^ |
| **TTA** | 234 | 1.74 | 1 | 0.57 |

Mutability index values were calculated by dividing observed number of mutations to expected number of mutations. The observed and expected numbers of mutations were compared by χ^2^ analysis and significant differences are indicated on mutability index values.

**^a^** statistically significant by χ^2^ test (*p* < 0.001)

**^b^** statistically significant by χ^2^ test (*p* < 0.01)

**^c^** statistically significant by χ^2^ test (*p* < 0.05)
