# Supplementary material for: T Cell Receptor Alpha Chain Genes in the Teleost Ballan Wrasse (Labrus bergylta) Are Subjected to Somatic Hypermutation
Source: Front Immunol. 2018 May 22;9:1101. doi: 10.3389/fimmu.2018.01101 (PMC5972329; doi:10.3389/fimmu.2018.01101)
Supplement: Supplementary file 10 [file table_10.docx]

**Supplementary TABLE 10. Accession numbers of clones used in the SHM studies of Ballan wrasse Vα and Cα**

| **Clone** | **Accession number** | **Clone** | **Accession number** |
| --- | --- | --- | --- |
| **Variable region** | | **Constant region** | |
| 04 | MG594749 | B1­-9 | MG594649 |
| 05 | MG594750 | B1­-13 | MG594700 |
| 09 | MG594752 | B1­-17 | MG594701 |
| 11 | MG594754 | B1­-22 | MG594652 |
| 12 | MG594755 | B1­-24 | MG594703 |
| 13 | MG594756 | B1­-28 | MG594653 |
| 14 | MG594757 | B1­-29 | MG594654 |
| 17 | MG594788 | B4-17 | MG594705 |
| 18 | MG594789 | B4-18 | MG594706 |
| 19 | MG594790 | B4-19 | MG594660 |
| 20 | MG594791 | B4-20 | MG594661 |
| 21 | MG594792 | B4-22 | MG594662 |
| 22 | MG594760 | B4-25 | MG594663 |
| 23 | MG594761 | B4-32 | MG594664 |
| 24 | MG594762 | B4-33 | MG594709 |
| 25 | MG594763 | B4-40 | MG594665 |
| 26 | MG594764 | B4-43 | MG594667 |
| 27 | MG594765 | B4-44 | MG594668 |
| 29 | MG594766 | B4-48 | MG594669 |
| 31 | MG594767 | B4-54 | MG594671 |
| 32 | MG594768 | B6-2 | MG594672 |
| 33 | MG594769 | B6-4 | MG594714 |
| 37 | MG594772 | B6-14 | MG594676 |
| 39 | MG594774 | B6-16 | MG594678 |
| 40 | MG594775 | B6-18 | MG594679 |
| 41 | MG594776 | B6-22 | MG594682 |
| 42 | MG594777 | B6-25 | MG594684 |
| 43 | MG594778 | B6-28 | MG594686 |
| 44 | MG594779 | B6-30 | MG594718 |
| 46 | MG594780 | B6-35 | MG594689 |
| 47 | MG594781 | B6-38 | MG594721 |
| 49 | MG594783 | B6-40 | MG594691 |
| 50 | MG594784 | B6-45 | MG594722 |
| 51 | MG594785 | B6-51 | MG594693 |
| 52 | MG594786 | B6-58 | MG594695 |
| 53 | MG594787 | B6-61 | MG594697 |
